# FASL rs763110 Polymorphism Contributes to Cancer Risk: An Updated Meta-Analysis Involving 43,295 Subjects

**DOI:** 10.1371/journal.pone.0074543

**Published:** 2013-09-23

**Authors:** Lei Xu, Xin Zhou, Feng Jiang, Man-Tang Qiu, Zhi Zhang, Rong Yin, Lin Xu

**Affiliations:** 1 The Fourth Clinical College of Nanjing Medical University, Nanjing, China; 2 Department of Thoracic Surgery, Nanjing Medical University Affiliated Cancer Hospital Cancer Institute of Jiangsu Province, Nanjing, China; 3 Department of Oncology, First Clinical College of Nanjing Medical University, Nanjing, China; The Institute of Cancer Research, United Kingdom

## Abstract

**Background:**

Published studies investigating the association between genetic polymorphism -884C/T (rs763110) of the FAS ligand (FASL) promoter and cancer risk reported inconclusive results. To derive a more precise estimation of the relationship, we performed an updated meta-analysis of all eligible studies.

**Methodology/Principal Findings:**

We carried out a meta-analysis, including 47 studies with 19,810 cases and 23,485 controls, to confirm a more conclusive association between the FASL rs763110 polymorphism and cancer susceptibility. Overall, significantly reduced cancer risk was associated with the variant -884T when all studies were pooled (TC vs. CC: OR = 0.83, 95%CI = 0.75–0.92; P_heterogeneity_<0.001; TT+TC vs. CC: OR = 0.85, 95%CI = 0.77–0.94; P_heterogeneity_<0.001). Stratified analysis revealed that there was a statistically reduced cancer risk in Asians (TC vs. CC: OR = 0.76, 95%CI = 0.67–0.87; P_heterogeneity_<0.001; TT+TC vs. CC: OR = 0.79, 95%CI = 0.70–0.90; P_heterogeneity_<0.001) and in patients with cancers of head and neck (TC vs. CC: OR = 0.87, 95%CI = 0.77–0.99; P_heterogeneity_ = 0.118; TT+TC vs. CC: OR = 0.88, 95%CI = 0.78–0.99; P_heterogeneity_ = 0.168) and ovarian cancer (TC vs. CC: OR = 0.67, 95%CI = 0.49–0.90; P_heterogeneity_ = 0.187; TT+TC vs. CC: OR = 0.64, 95%CI = 0.48–0.86; P_heterogeneity_ = 0.199). Meta-regression showed that ethnicity (p = 0.029) and genotyping method (p = 0.043) but not cancer types (p = 0.772), sample size (p = 0.518), or source of controls (p = 0.826) were the source of heterogeneity in heterozygote comparison.

**Conclusion:**

Our results suggest that the FASL polymorphism rs763110 is associated with a significantly reduced risk of cancer, especially in Asian populations.

## Introduction

Cancer is a major public health burden all around the world which counts for one in 4 deaths in the United States [1]. The global burden of cancer continues to increase largely because of the aging and growth of the world population as well as an increasing adoption of cancer-related lifestyle, such as smoking, physical inactivity and ‘‘westernized’’ diets [2]. It was reported that there was about 12.7 million new cancer cases and 7.6 million cancer deaths throughout the world in 2008 [3]. However, the mechanism of carcinogenesis is complicated and remains largely unknown. Many studies identified that genetics play a vital role in determining cancer risk and various genetic variations have been identified to elevate cancer risk [4]. Single nucleotide polymorphisms (SNPs) are the most common form of human genetic variation and may contribute to individual’s cancer risk through interaction with environmental factors [5] .

Apoptosis plays an important role in various physiological functions and pathological processes, including immune diseases and carcinogenesis [6], [7], and defects in apoptotic pathways are suggested to be associated with a number of human diseases, ranging from neurodegenerative disorders to various cancers [8]. FASL is a transmembrane protein belonging to the tumor necrosis factor (TNF) superfamily which can trigger apoptotic cell death by ligation to its receptor, Fas (CD95/APO-1). Numerous evidence suggested that FASL could mediate immune privilege in human tumors by inducing FAS-mediated apoptosis in tumor-specific lymphocytes [9].

Recently many common low-penetrance genes have been considered as potential markers of cancer susceptibility. FASL gene is an important one of them, which situated on chromosome 1q23 with four exons. Though it is highly polymorphic, but the polymorphism C to T substitution at position -844(FASL-844C/T, rs763110) in the promoter region has been studied extensively [10]. It is located in a binding motif for another transcription factor, CAAT/enhancer-binding proteinβand a higher basal expression of FASL is significantly associated with the FASL -844C allele compared with the - 844T allele [11].

In the past decade, numerous studies have suggested that the FASL -844C/T polymorphism is associated with many types of cancers [12]–[55], but the results are conflicting rather than conclusive. Although two meta-analyses have discussed the rs763110 polymorphism and susceptibility to cancers [56], [57], but they did not include all of the eligible studies, especially the case-control studies published in the past five years. Therefore, we performed this updated meta-analysis of 47 association studies of the FASL rs763110 polymorphism and cancer risk (including a total of 43,295 participates, approximately twice as many subjects as in previous such meta-analysis).

## Methods

### Publication search

PubMed and China National Knowledge Infrastructure (CNKI) were searched comprehensively using the terms relating to the FASL gene (e.g. “FASL”, “FAS ligand” or “CD95L”) in combination with words related to cancer (e.g. “cancer”, “carcinoma”, “tumor” or “neoplasm”) and polymorphism or variation. Last search was updated on May 29, 2013.

In order to minimize potential publication bias, there were no language and other restrictions. Furthermore, citations in the retrieved articles were manually examined to identify additional relevant studies. Only the most recent or complete study was used if more than one of the same patient populations was applied in several publications.

### Inclusion and exclusion criteria

The major inclusion criteria were: (1) case-control or nested case-control studies; (2) investigating the association between the FASL rs763110 polymorphism and cancer risk; (3) cancers diagnosed by histopathology; (4) sufficient data for calculating an odds ratio (OR) with 95% confidence intervals (CIs). Accordingly, case-only studies, reviews and repeated papers were excluded.

### Data extraction

Data were independently extracted by two reviewers (Xu and Zhou) and checked by the other authors. The following information was abstracted: name of the first author, year of publication, country where the study was conducted, genotyping method, ethnicity, cancer types, source of controls, age, gender, number of cases and controls, genotype frequency in cases and controls and Hardy-Winberg equilibrium (HWE). Different ethnicities were classified as Caucasian, Asian, and African. All eligible studies were defined as hospital-based (HB) or population-based (PB) according to the source of controls. In case of discrepancies, a consensus on each item was reached among the authors.

### Statistical analysis

For the controls of each study, Hardy-Winberg equilibrium (HWE) was evaluated using the goodness-of-fit chi-square test and a p<0.05 was considered with a significant selective bias [58]. Crude ORs with 95% CIs were used to assess the strength of association between the FALS rs763110 polymorphism and cancer susceptibility and a 95% CI without 1 for OR indicating a significantly increased or reduced cancer risk. The pooled ORs were performed for homozygote comparison (TT versus CC), heterozygote comparison (TC versus CC), dominant (TC+TT versus CC) and recessive (TT versus TC+CC) modes, respectively. Subgroup analyses were also performed to investigate the effects of confounding factors: cancer types, ethnicities, sample size (studies with more than 1000 subjects were sorted as “large”, and studies with less than 1000 subjects were sorted as “small”) and source of controls. Sensitivity analyses were conducted to identify individual study’ effect on pooled results and test the reliability of results. Heterogeneity assumption was checked by the chi-square based Q test, and the heterogeneity was considered significant when p<0.10 [59]. The random-effects model (based on DerSimonian-Laird method) was used when heterogeneity existed among studies; otherwise the fixed-effects model (based on Mantel-Haenszel method) was applied [60]. Stratification and meta-regression analyses were used to detect the potential heterogeneity among studies. The presence of publication bias was examined by Begg’s funnel plot and the Egger’ linear regression test, and a p<0.05 was considered significant [61]. All statistical analyses were performed with STATA software (version 12.0; StataCorp, College Station, Texas USA). And all P values were two-side.

## Results

### Characteristics of eligible studies

**Figure 1:**
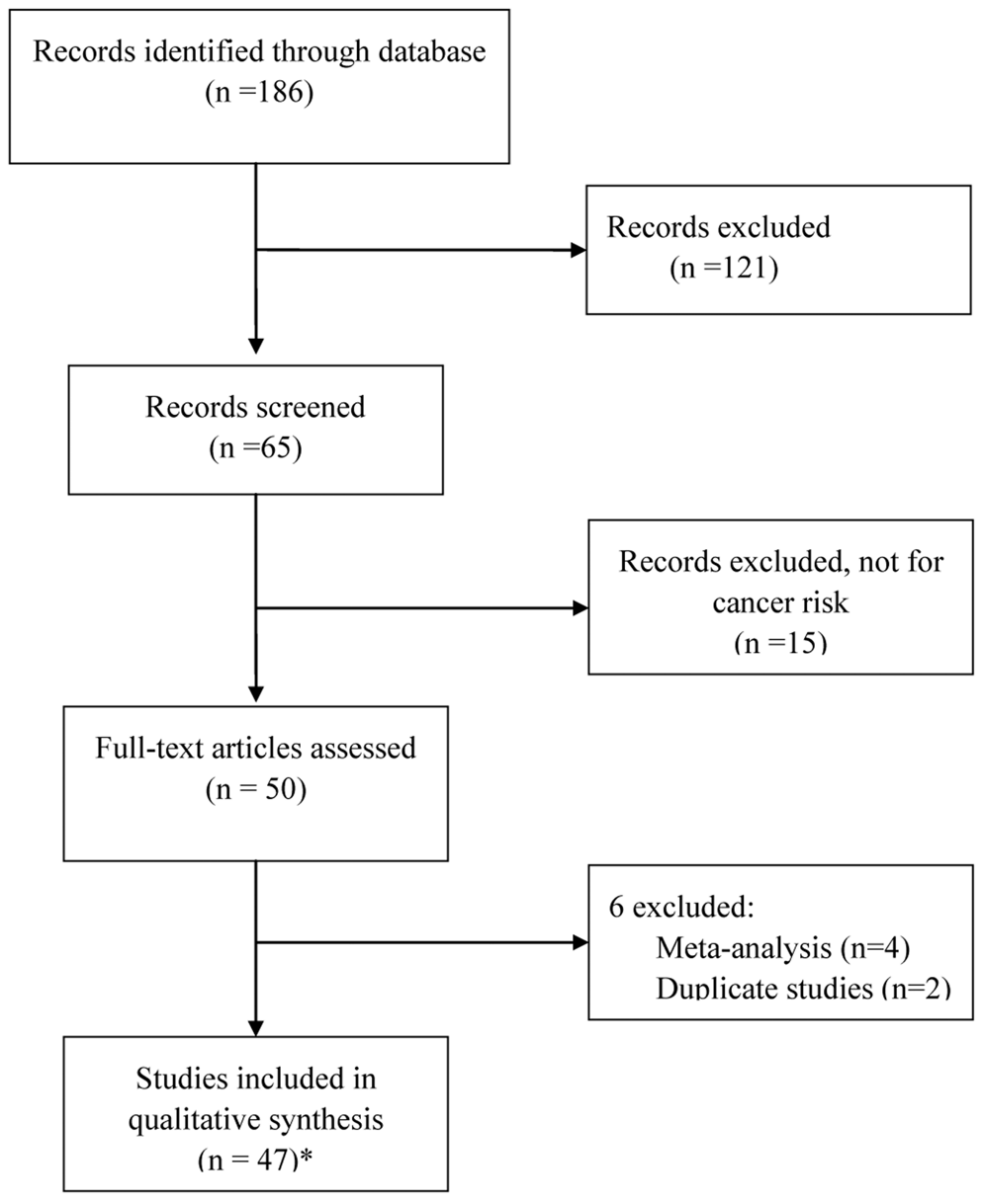
PRISMA Flow Chart. *a total of 44 articles were identified and five separate studies were reported in two articles, thus 47 studies were eligible

After careful retrieve and selection, 44 articles (listed in Table 1) were identified according to inclusion and exclusion criteria. The flow chart of selection was shown in Figure 1. Qureshi’s and Chatterjee’s studies sorted the data into three types of cancers and two ethnicities respectively. Each group in these studies was considered separately. Thus, a total of 47 case-control studies, including 19,810 cases and 23,485 controls were analyzed in this meta-analysis.

**Table 1:** Main characteristics of eligible studies.

Author	Year	Country	Ethnicity	Cancer type	Study design	Method	Cases	Controls	Phwe
Sun	2004	China	Asian	Esophageal	PB	PCR-RFLP	588	648	0.061
Krippl	2004	Austria	Caucasian	Breast	PB	Taqman	489	487	0.418
Lai	2005	China	Asian	Cervical	HB	Taqman	303	316	0.920
Sun	2005	China	Asian	Cervical	PB	PCR-RFLP	314	615	0.002 *
Zhang	2005	China	Asian	Lung	PB	PCR-RFLP	1000	1270	0.180
Yang	2005	China	Asian	colorectal	PB	PCR-RFLP	382	648	0.061
Park	2006	Korea	Asian	Lung	HB	PCR-RFLP	582	582	0.570
Li	2006	China	Asian	Bladder	HB	PCR-RFLP	216	252	0.234
Zhang	2006	USA	Caucasian	SCCNH	HB	PCR-RFLP	721	1234	0.411
Li	2006	USA	Caucasian	Melanoma	HB	PCR-RFLP	602	603	0.071
Zhang	2007	China	Asian	Breast	PB	PCR-RFLP	839	830	0.110
Erdogan	2007	Turkey	Caucasian	Thyroid	HB	PCR-RFLP	45	100	0.727
Gormus	2007	Turkey	Caucasian	Ovarian	HB	PCR-RFLP	47	41	0.678
Crew	2007	USA	Caucasian	Breast	PB	Taqman	1062	1105	0.602
Ivansson	2007	Sweden	Caucasian	Cervical	PB	Taqman	1284	280	0.738
Zhang	2007	Sweden	Caucasian	Melanoma	PB	PCR-RFLP	229	351	0.609
Kang	2008	Korea	Asian	Cervical	HB	PCR-RFLP	154	160	0.327
HSU	2008	China	Asian	Gastric	HB	PCR-RFLP	86	101	0.612
Yang	2008	China	Asian	Pancreatic	PB	PCR-RFLP	397	907	0.986
Ter-Minassi	2008	USA	Caucasian	Lung	HB	Taqman	2147	1490	0.254
Chatterjee	2009	South Africa	African	Cervical	HB	Taqman	103	100	0.469
Chatterjee	2009	South Africa	African	Cervical	HB	Taqman	327	315	0.457
Wang	2009	China	Asian	Gastric	HB	PCR-RFLP	332	324	0.554
Chen	2009	China	Asian	Esophageal	PB	PCR-RFLP	188	324	0.464
Zhang	2010	China	Asian	Esophageal	HB	PCR-RFLP	204	248	0.254
Zhou	2010	China	Asian	Cardiac	HB	PCR-RFLP	262	524	0.899
Liu	2010	China	Asian	Cardiac	HB	PCR-RFLP	344	324	0.083
Zhu	2010	China	Asian	Renal	HB	Taqman	353	365	0.278
Kim	2010	Korea	Asian	AML	PB	PCR-RFLP	590	858	0.076
Wang	2010	China	Asian	OSCC	PB	PCR-RFLP	294	333	0.271
Cao	2010	China	Asian	Nasopharyngeal	PB	PCR-RFLP	563	610	0.004 *
Zhai	2010	USA	Caucasian	Esophageal	HB	PCR-RFLP	305	339	0.388
Qureshi	2010	USA	Caucasian	Melanoma	PB	PCR-RFLP	217	852	0.427
Qureshi	2010	USA	Caucasian	SCC	PB	PCR-RFLP	278	852	0.427
Qureshi	2010	USA	Caucasian	BCC	PB	PCR-RFLP	286	852	0.427
Zhang	2011	China	Asian	Gastric	HB	PCR-RFLP	234	321	0.094
Shao	2011	China	Asian	Prostate	HB	PCR-RFLP	602	703	0.801
Kupcinska s	2011	Germany	Caucasian	Gastric	HB	Taqman	114	238	0.715
Mahfoudh	2012	Tunisia	African	Breast	PB	PCR-RFLP	438	332	0.334
Tong	2012	China	Asian	ALL	HB	PCR-RFLP	361	519	0.137
Wang	2012	China	Asian	Breast	HB	PCR-RFLP	420	496	0.112
Zhang	2012	China	Asian	Cardiac	HB	PCR-RFLP	375	496	0.112
Liu	2012	China	Asian	Gastric	HB	PCR-RFLP	218	218	0.073
Li	2012	China	Asian	Ovarian	HB	ASMLDR	342	344	0.547
Karimi	2012	India	Asian	OSCC	PB*	PCR-RFLP	139	126	0.514
Hashemi	2013	Iran	Asian	Breast	PB	T-ARMS-PCR	134	152	0.184
Wang	2013	China	Asian	LHSCC	PB	PCR-RFLP	300	300	0.990

OSCC: oral squamous cell carcinoma; SCC: squamous cell carcinoma of skin; BCC: basal cell carcinoma of skin; SCCNH: squamous cell carcinoma of the head and neck; LHSCC: larynx and hypopharynx squamous cell carcinoma; ALL: acute lymphoblastic leukemia AML: acute myeloid leukemia PB: population-based; HB: hospital-based Phwe: Hardy-Winberg equilibrium; PB*: not defined; Phwe*<0.05 ASMLDR: allele-specific multiple ligase detection reactions; T-ARMS-PCR: Tetra-amplification refractory mutation system–polymerase chain reaction; PCR-RFLP: Polymerase chain reaction-restriction fragment length polymorphism

Of the 47 studies, 42 were published in English and 5 in Chinese, 14 of them were studies of Caucasians, 30 studies of Asian and 3 studies of African (details shown in Table 1). All cases were histopathologically confirmed. Controls were mainly matched for age and/or gender, of which 21 were population-based (PB) and 26 were hospital-based (HB). One of the studies did not show source of controls, we considered it to be population-based [15]. All studies showed that the distribution of genotypes in the control group was in agreement with the Hardy-Winberg equilibrium (HWE) except for two studies (Cao [23], p = 0.004 and Sun [48], p = 0.002).

### Main results

**Figure 2:**
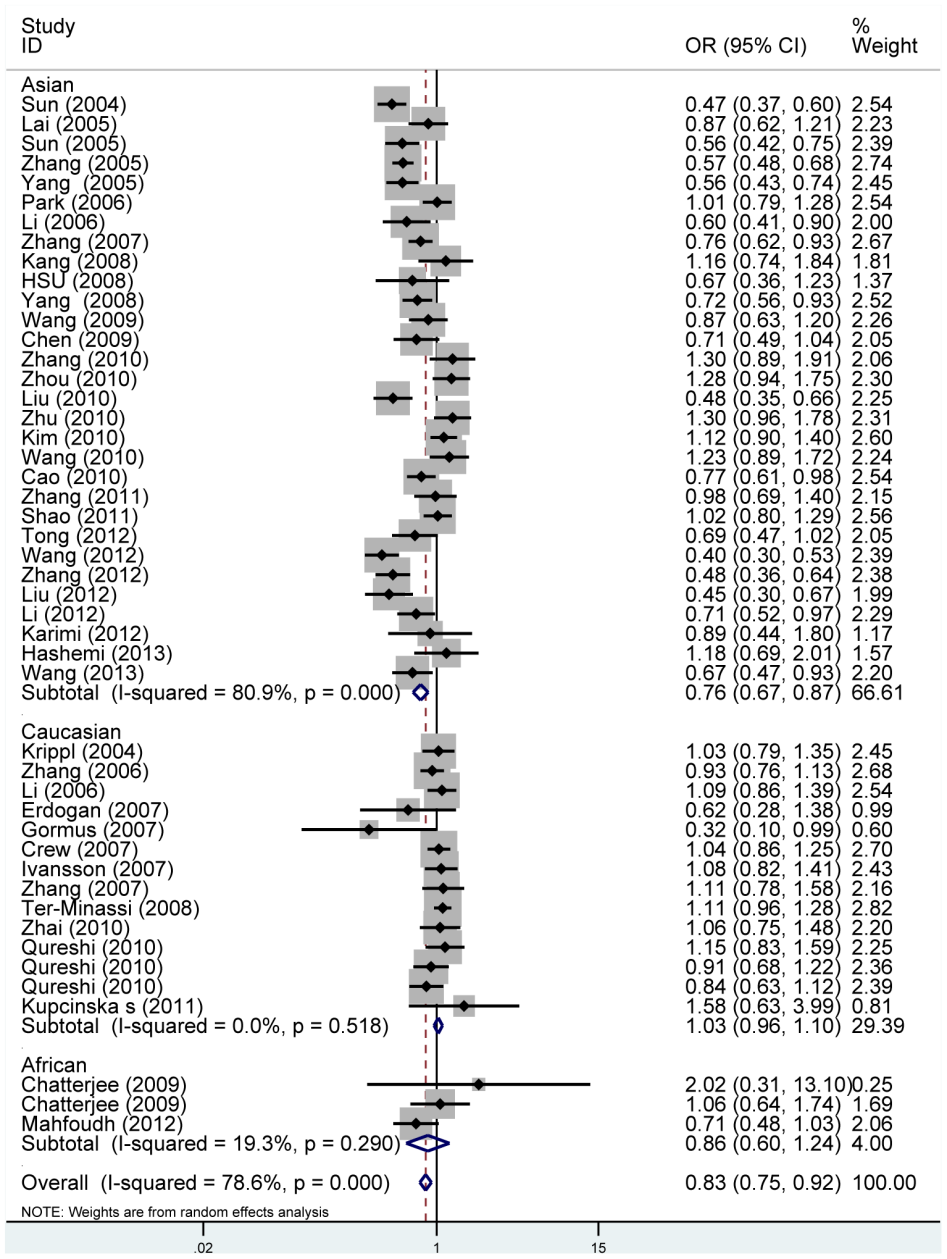
Subgroup analysis by ethnicity of ORs with a random-effects model for associations between the FASL rs763110 polymorphism and cancer risk under dominant model (TC+TT vs. CC).

**Figure 3:**
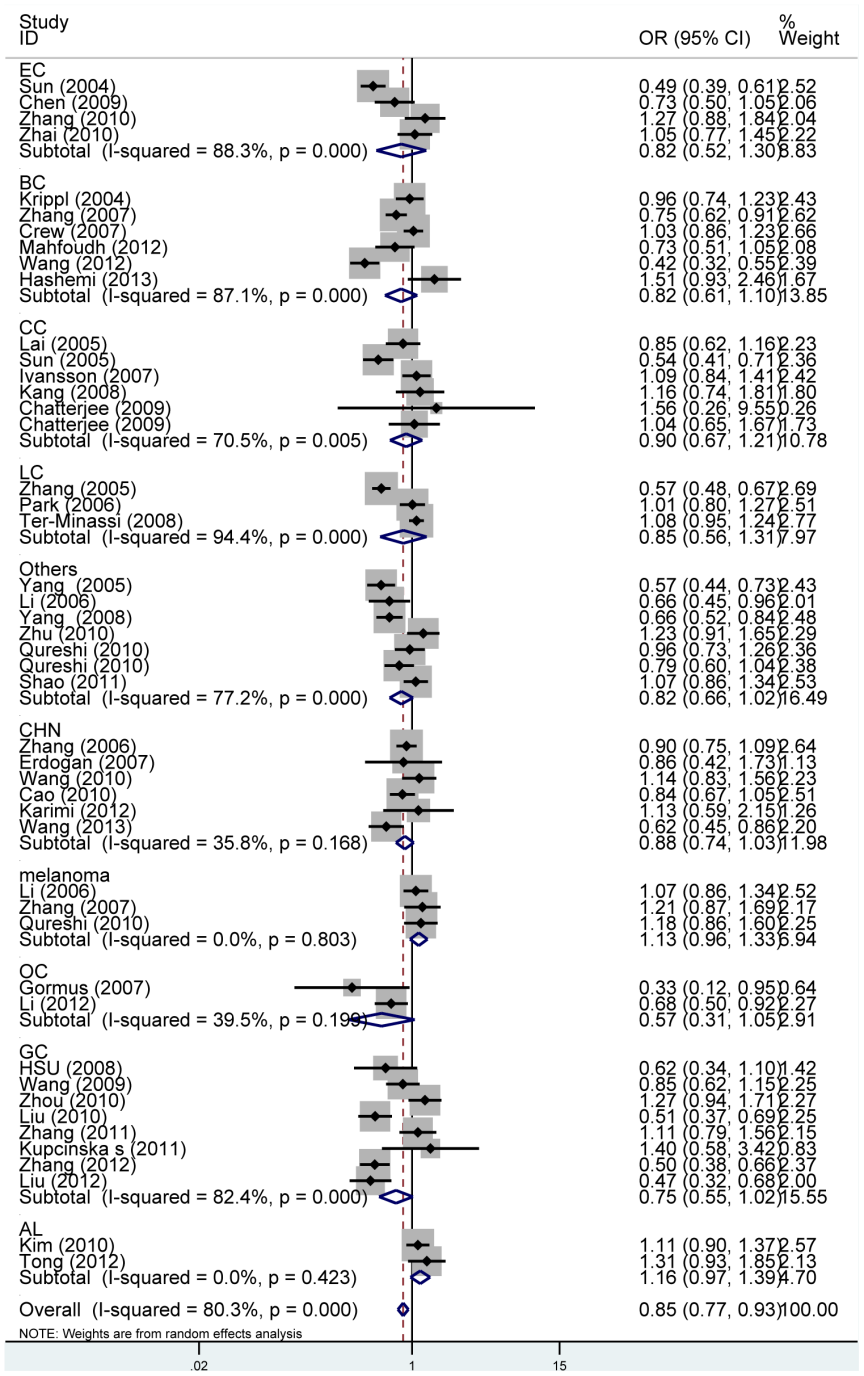
Subgroup analysis by cancer type of ORs with a random-effects model for associations between the FASL rs763110 polymorphism and cancer risk under heterozygote comparison (TC vs. CC). BC: breast cancer; LC: lung cancer; EC: esophageal cancer; CC: cervical cancer GC: gastric cancer & cardiac cancer AL: acute lymphoblastic leukemia & acute myeloid leukemia OC: ovarian cancer CHN: cancers of head and neck

Table 2 showed the main results of this meta-analysis. Overall, significantly reduced cancer risk was associated with the FASL -844T allele when all studies were pooled (TC vs. CC: OR = 0.83, 95%CI = 0.75–0.92; P_heterogeneity_<0.001, Figure 2; dominant model: OR = 0.85, 95%CI = 0.77–0.94; P_heterogeneity_<0.001, Figure 3). No significant association was found in homozygote comparison (TT vs. CC: OR = 0.89, 95%CI =  0.79–1.01; P_heterogeneity_ = 0.074) or recessive model (TT vs. TC+CC: OR =  0.97, 95%CI =  0.86–1.09; P_heterogeneity_<0.001).

**Table 2:** Meta-analysis results.

	n	TT vs. CC	Ph	TC vs. CC	Ph	TT+TC vs. CC	Ph	TT vs. TC+CC	Ph
Total	47	0.89(0.79,1.01)	0.074	0.83(0.75,0.92)*	<0.001	0.85(0.77,0.94)*	<0.001	0.97(0.86,1.09)	<0.001
Cancer type									
EC	4	0.79(0.6,1.04)^a^	0.308	0.82(0.5,1.33)	<0.001	0.82(0.52,1.3)	<0.001	0.9(0.69,1.17)^a^	0.889
LC	3	0.84(0.58,1.23)	0.012	0.86(0.55,1.33)	<0.001	0.85(0.56,1.31)	<0.001	0.91(0.79,1.06)^a^	0.292
BC	6	0.85(0.62,1.16)	0.003	0.79(0.59,1.07)	<0.001	0.82(0.61,1.1)	<0.001	0.94(0.74,1.19)	0.029
CC	6	0.86(0.66,1.11)^a^	0.165	0.91(0.69,1.21)	0.013	0.90(0.67,1.21)	<0.001	0.89(0.72,1.08)^a^	0.445
Others	7	0.8(0.55,1.15)	0.003	0.83(0.67,1.02)	0.001	0.82(0.66,1.02)	<0.001	0.87(0.63,1.20)	0.011
Melanoma	3	1.22(0.93,1.59)^a^	0.277	1.11(0.94,1.32)^a^	0.968	1.13(0.96,1.33)^a^	0.803	1.15(0.89,1.49)^a^	0.272
CHN	6	0.94(0.66,1.33)	0.041	0.87(0.77,0.99)*^a^	0.118	0.88(0.78,0.99)^*a^	0.168	1.04(0.71,1.5)	0.008
GC	8	0.85(0.6,1.2)	0.038	0.73(0.53,1.01)	<0.001	0.75(0.55,1.02)	<0.001	0.94(0.76,1.15)^a^	0.234
AL	2	1.66(0.69,4.01)	0.001	0.91(0.57,1.45)	0.032	1.16(0.97,1.39)^a^	0.423	1.84(0.57,5.95)	<0.001
OC	2	0.48(0.27,0.87)*^a^	0.547	0.67(0.49,0.9)*^a^	0.187	0.64(0.48,0.86)*^a^	0.199	0.65(0.38,1.11)^a^	0.744
Source of control									
PB	21	0.84(0.7,1)	<0.001	0.82(0.72,0.93)*	<0.001	0.83(0.73,0.95)*	<0.001	0.92(0.79,1.07)	<0.001
HB	26	0.95(0.80,1.13)	<0.001	0.84(0.73,0.97)*	<0.001	0.87(0.76,0.99)*	<0.001	0.97(0.86,1.09)	<0.001
Ethnicity									
Asian	30	0.83(0.68,1.02)	<0.001	0.76(0.67,0.87)*	<0.001	0.79(0.7,0.9)*	<0.001	0.95(0.77,1.16)	<0.001
Caucasian	14	0.98(0.89,1.09)^a^	0.182	1.03(0.96,1.1)^a^	0.518	1.02(0.95,1.09)^a^	0.399	0.96(0.88,1.06)^a^	0.335
African	3	0.88(0.64,1.2)^a^	0.595	0.84(0.62,1.13)^a^	0.290	0.85(0.64,1.12)^a^	0.396	0.95(0.78,1.17)^a^	0.700
Sample size									
Large	17	0.88(0.75,1.02)	<0.001	0.86(0.76,0.98)*	<0.001	0.86(0.76,0.98)*	<0.001	0.93(0.83,1.04)	0.043
Small	30	0.91(0.74,1.11)	<0.001	0.81(0.7,0.93)*	<0.001	0.84(0.73,0.97)*	<0.001	0.99(0.813,1.21)	<0.001
Genotyping method									
PCR-RFLP	36	0.86(0.74,1.01)	<0.001	0.79(0.70,0.87)*	<0.001	0.80(0.72,0.90)*	<0.001	0.98(0.84,1.15)	<0.001
Taqman	9	0.98(0.86,1.11)^a^	0.855	1.08(0.99,1.18)^a^	0.801	1.05(0.97,1.14)^a^	0.803	0.93(0.83,1.03)^a^	0.855

N: number of studies included; OR: odds ratio; Ph: p value for heterogeneity; BC: breast cancer; LC: lung cancer; EC: esophageal cancer; CC: cervical cancer GC: gastric cancer & cardiac cancer AL: acute lymphoblastic leukemia & acute myeloid leukemia OC: ovarian cancer CHN: cancers of head and neck PB: population-based; HB: hospital-based; *OR with statistical significance; large: studies with more than 1000 participants; small: studies with less than 1000 participants; PCR-RFLP: Polymerase chain reaction-restriction fragment length polymorphism; ^a^OR: estimates for fixed effects model.

In the sub-group analyses by ethnicity, significant reduced risks were found for T carriers among Asians (TC vs. CC: OR = 0.76, 95%CI = 0.67–0.87; P_heterogeneity_<0.001, Figure 2; dominant model: OR = 0.79, 95%CI = 0.70–0.90; P_heterogeneity_<0.001). In Caucasians and Africans, however, no significant association was found in each comparison.

When we performed sub-group analyses by cancer types, reduced cancer risk was found in the heterozygote and dominant model comparison for cancers of head and neck ( TC vs. CC: OR = 0.87, 95%CI = 0.77–0.99; P_heterogeneity_ = 0.118; dominant model: OR = 0.88, 95%CI = 0.78–0.99; P_heterogeneity_ = 0.168, Figure 3) and ovarian cancer (TC vs. CC: OR = 0.67, 95%CI = 0.49–0.90; P_heterogeneity_ = 0.187; TT+TC vs. CC: OR = 0.64, 95%CI = 0.48–0.86; P_heterogeneity_ = 0.199, Figure 3), respectively.

The results of the sub-group analyses by sample size, source of control and genotyping method were shown in supplemental information (Figure S1 – S3 in File S1). We found a statistically significant link between the FASL rs763110 polymorphism and cancer risk in studies utilizing genotyping method with polymerase chain reaction-restriction fragment length polymorphism (PCR-RFLP) assay in heterozygote and dominant model comparison (TC vs. CC: OR = 0.79, 95%CI = 0.70–0.87; P_heterogeneity_<0.001; dominant model: OR = 0.80, 95%CI = 0.72–0.90; P_heterogeneity_<0.001), but not for studies using Taqman assay.

### Heterogeneity

Heterogeneity among studies was identified in overall comparisons and also in sub-group analyses (shown in Table 2). To examine the potential source of heterogeneity, meta-regression was performed using variables as cancer type, source of control, ethnicity, genotyping method and sample size in heterozygote comparison (TC vs. CC). The results revealed that ethnicity (p = 0.029) and genotyping method (p = 0.043) but not cancer types (p = 0.772), sample size (p = 0.518), or source of controls (p = 0.826) contributed to the source of heterogeneity.

### Sensitivity analysis

Leave-one-out sensitivity analysis was performed to explore individual study’s influence on the pooled results. The results revealed that no individual study affected the pooled OR significantly since no substantial change was found (figure not shown).

### Publication bias

**Figure 4:**
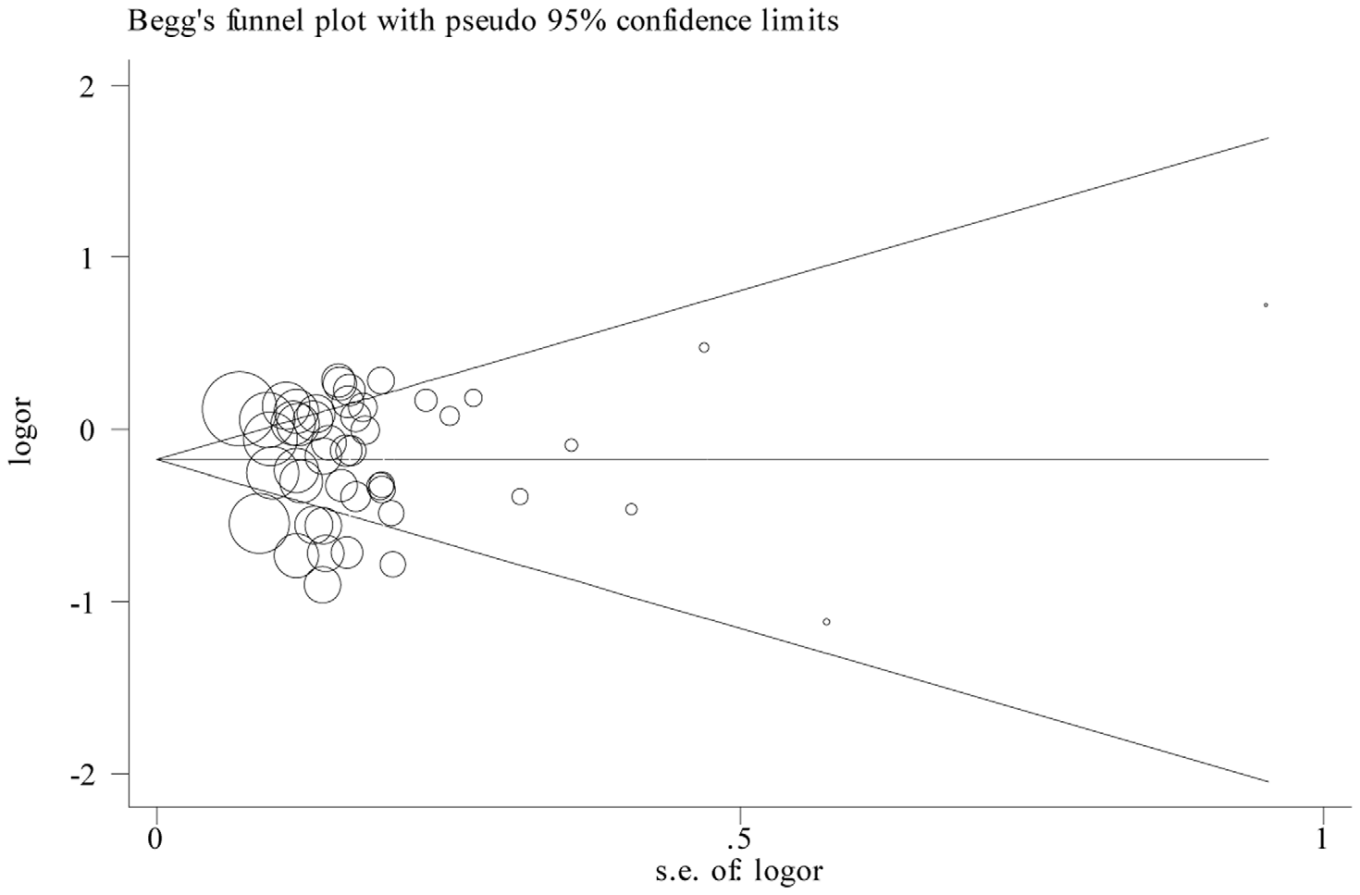
Funnel plot of heterozygote comparison (TC vs. CC). Funnel plot of all 47 eligible studies p =  0.430, Egger’s test p = 0.02; the circles represent the weight of individual study.

Begg’s funnel plot and Egger’s test were performed to assess the publication bias. The figure of the funnel plot did not show any evidence of obvious asymmetry (p = 0.430 for TC vs. CC) (Figure 4). Then, the Egger’s test was used to statistical test and publication bias was not detected either (p = 0.572 for TT vs. CC, p = 0.714 for TC vs. CC, p =  0.967 for dominant model, and p = 0.388 for recessive model, respectively).

## Discussion

A total of 47 eligible studies, including 19,810 cases and 23,485 controls were identified and analyzed in this meta-analysis. We demonstrated that the FASL -844T allele was associated with a statistically reduced risk of cancer. This significant association was found in Asians but not for Caucasians or Africans. With all published data, this finding might be plausible.

All controls in the studies involved were mainly cancer-free. The distribution of genotype in the control group was consistent with the Hardy-Weinberg equilibrium in all studies except for two studies (shown in Table 1). When excluding these two studies, the pooled OR and heterogeneity were not significantly changed indicating that the control group could represent the base population.

The FAS/FASL signaling system plays an important role in the cell apoptosis pathway, including regulation of immune system, maintaining immune-privileged ability and performing other regulatory functions [62], [63]. FAS is a cell surface receptor which expresses in various tissues [64], and FASL is the natural ligand to FAS [65]. The FAS combination with the FASL may trigger the death signal cascade, and subsequently leads cells to die. It has been reported that the aberration of expression of FAS and/or FASL results in cancer cells resisting the killing of T lymphocytes and is related to many human tumors [66], [67]. SNPs in a gene may influence its transcription or translation and eventually alter the biological function. The most popular polymorphism for FASL is a C to T changes at nucleotide position -844(rs763110) in the promoter region which may influence FASL expression, apoptosis signaling pathway, and ultimately contribute to the susceptibility to cancer. Numerous researches have proved that the FASL rs763110 polymorphism was associated with cancer risk (studies listed in Table 1). However, the results were controversial. Although meta-analyses of this polymorphism have been performed by the former scholars, in our present study, much more data were included and may get more comprehensive information.

With newly added studies, we carried out sub-group analyses. When stratified by ethnics, we found a significant association in Asians, but not for Caucasians or Africans, which may suggest that ethnic variation of genetic background would be modified by environmental factors [68], such as age, sex, diet, lifestyle, smoking, BMI, and so on. In our study, the frequency of the FASL-844T alleles was 28.4%, 36.9%, and 62.8% in Asians, Caucasians, and Africans respectively, and the -844 C to T mutant rate among them maybe different, which may count for the results stratified by ethnics. Furthermore, it was reported that studies with small size may have insufficient statistical power to investigate a slight effect on the pooled results or may produce a fluctuated risk estimate, which may cause the ethnic differences [69]. We could find that the participants of the three ethnicities differ greatly from each other in Table 2.

In the sub-group analysis by cancer type, no significant association was found except for heterozygote and dominant model comparison of ovarian cancer and cancers of head and neck (shown in Table 2). In our study, the cancers of head and neck subgroup consisted of cancers of oral cavity, pharynx and larynx, thyroid, and nasopharynx, and most of them were squamous cell carcinoma. Gastman and colleagues reported that the FAS/FASL pathway may participate in the immunosuppression process in head and neck cancer [70]. Based on our findings, we speculate that the -844C/T rs763110 polymorphism of FASL may be a potential genetic biomarker for risk of head and neck cancer. As for the significance of ovarian cancer, the relatively small sample size may weaken the statistical power and lead to the results considerable, so many case-control studies with more participants investigating ovarian cancer and the -844C/T rs763110 polymorphism are needed to prove the result. Different cancer risks were also found in the studies using different genotyping methods. We discovered that the association was significant among studies utilizing PCR-RFLP assay, but not for studies with Taqman genotyping assay. This may be explained that most studies for Asians utilizing PCR-RFLP, nevertheless Taqman was the main genotyping method for Caucasian and African studies.

Attention should be paid to the relatively large heterogeneity in our results. Meta-regression was performed for heterozygote model according to ethnicity, cancer type, source of control, genotyping method and sample size. We found the sources of heterogeneity were mainly from ethnicity (p = 0.029) and genotyping method (p = 0.043). In addition, through sub-group analysis by ethnicity, we found that I-squared for the Asian, Caucasian and African studies was 80.9%, 0.0% and 19.3%, respectively (Figure 2). Then we demonstrated that the heterogeneity might mainly come from the Asian studies. In fact, many other factors may also be the potential source of heterogeneity. Due to lack of detailed data, we had to give up performing a meta-regression utilizing these variables.

Some limitation should be noted in this meta-analysis. Firstly, the controls were not uniformly defined and some studies included inpatient with benign disease which may contribute to the FASL gene mutation and development of various cancers. Secondly, due to limited individual data, we did not conduct a more precise analysis on other covariates such as age, gender, and environmental factors. Thirdly, the heterogeneity is difficult to exclude, in that it is influenced by complicated factors, such as age, sex, genetic diversities, different lifestyle, and clinical characteristics.

Despite these limitations, our meta-analysis had significantly higher statistical power than the previous study that analyzed the association between the FASL -844C/T rs763110 polymorphism and cancer risk, since the cancer patients involved in our meta-analysis were twice as many as the previous one. We also analyzed the rs763110 polymorphism in various populations, including Africans. Cancer types in our study were more multifarious and a significant association was also found in cancers of head and neck and ovarian cancer though the sample size was relatively small.

In conclusion, this meta-analysis suggests that the FASL-844C/T rs763110 polymorphism is associated with a significantly reduced risk of cancer, especially in Asian populations. To verify these results, large scale case-control studies with detailed individual information are needed.

## Supporting Information

Checklist S1 **PRISMA checklist.**
(DOC)

File S1 **Subgroup analysis by sample size, source of control and genotyping method of ORs with a random-effects model for associations between FASL rs763110 polymorphism and cancer risk under heterozygote comparison (TC vs. CC).**
(DOCX)
