# Correction: FASL rs763110 Polymorphism Contributes to Cancer Risk: An Updated Meta-Analysis Involving 43,295 Subjects

**DOI:** 10.1371/annotation/6f1720b5-73e8-428d-87f5-3eb671c77a98

**Published:** 2013-10-24

**Authors:** Lei Xu, Xin Zhou, Feng Jiang, Man-Tang Qiu, Zhi Zhang, Rong Yin, Lin Xu

The sixth author, Rong Yin, was not correctly indicated in the byline as a Corresponding Author for the article. The six author's email address is: yinhero001@126.com.

In addition, the email address of the other Corresponding Author, seventh author, Lin Xu, is incorrect. The seventh author's correct email address is: xulin83cn@gmail.com. 

