# Supplementary material for: FASL rs763110 Polymorphism Contributes to Cancer Risk: An Updated Meta-Analysis Involving 43,295 Subjects
Source: PLoS One. 2013 Sep 23;8(9):e74543. doi: 10.1371/journal.pone.0074543 (PMC3781150; doi:10.1371/journal.pone.0074543)
Supplement: File S1 — Subgroup analysis by sample size, source of control and genotyping method of ORs with a random-effects model for associations between FASL rs763110 polymorphism and cancer risk under heterozygote comparison (TC vs. CC). (DOCX) [file pone.0074543.s002.docx]

Figure S1: Subgroup analysis by sample size of ORs with a random-effects model for associations between the FASL rs763110 polymorphism and cancer risk under heterozygote comparison (TC vs. CC).

Figure S2: Subgroup analysis by source of control of ORs with a random-effects model for associations between the FASL rs763110 polymorphism and cancer risk under heterozygote comparison (TC vs. CC).

Figure S3: Subgroup analysis by genotyping method of ORs with a random-effects model for associations between the FASL rs763110 polymorphism and cancer risk under heterozygote comparison (TC vs. CC).
